# Venous Outflow Reconstruction in Adult Living Donor Liver Transplant: Outcome of a Policy for Right Lobe Grafts without the Middle Hepatic Vein

**DOI:** 10.1155/2013/280857

**Published:** 2013-12-30

**Authors:** Mohamed Ghazaly, Mohamad T. Badawy, Hosam El-Din Soliman, Magdy El-Gendy, Tarek Ibrahim, Brian R. Davidson

**Affiliations:** ^1^Liver Transplant Department, National Liver Institute, Menoufiya University, Shebeen El-Koum, Egypt; ^2^University Department of Surgery and Liver Transplant Unit, Royal Free Hospital Trust and Royal Free and University College School of Medicine, Hampstead Campus, Pond Street, London NW3 2QG, UK

## Abstract

*Introduction*. The difficulty and challenge of recovering a right lobe graft without MHV drainage is reconstructing the outflow tract of the hepatic veins. With the inclusion or the reconstruction of the MHV, early graft function is satisfactory. The inclusion of the MHV or not in the donor's right lobectomy should be based on sound criteria to provide adequate functional liver mass for recipient, while keeping risk to donor to the minimum. *Objective*. Reviewing the results of a policy for right lobe grafts transplant without MHV and analyzing methods of venous reconstruction related to outcome. *Materials and Methods*. We have two groups Group A (with more than one HV anast.) (*n* = 16) and Group B (single HV anast.) (*n* = 24). Both groups were compared regarding indications for reconstruction, complications, and operative details and outcomes, besides describing different modalities used for venous reconstruction. *Results*. Significant increase in operative details time in Group A. When comparison came to complications and outcomes in terms of laboratory findings and overall hospital stay, there were no significant differences. Three-month and one-year survival were better in Group A. *Conclusion*. Adult LDLT is safely achieved with better outcome to recipients and donors by recovering the right lobe without MHV, provided that significant MHV tributaries (segments V, VIII more than 5 mm) are reconstructed, and any accessory considerable inferior right hepatic veins (IRHVs) or superficial RHVs are anastomosed.

## 1. Introduction

Chronic liver disease and cirrhosis are important causes of morbidity and mortality in the world. Moreover, the burden of chronic liver disease is projected to increase due in part to the increasing prevalence of end-stage liver disease and HCC secondary to NAFLD and HCV. Liver transplantation is the best treatment option for end-stage liver disease, including early HCC associated with advanced cirrhosis. However, the application of liver transplantation is severely limited by the shortage of deceased donor grafts; hence many patients die from progression of the disease while waiting for a graft [1].

The shortage of cadaveric livers has sparked an interest in living donor liver transplantation (LDLT). LDLT may increase the liver graft pool and reduce waiting list mortality [2, 3]. In adults, right hemiliver graft can satisfy the demands of the recipient's metabolism and prevent small-for-size syndrome. The difficulty and challenge of LDLT without MHV drainage is providing adequate venous drainage of the graft [4, 5]. Obstruction of venous outflow leads to graft congestion and failure [6].

The major controversy with right lobe LDLT lies in the necessity for including the MHV in the graft and in concerns for the safety of the donor. The MHV carries out important venous drainage for the right anterior segment and is essential for perfect graft function in nearly 85% of right lobe LDLTs [7]. In the absence of the MHV, the right anterior segment of the liver graft may suffer from congestion and damage with subsequent diffuse mechanical injury to the right posterior segment, and the liver graft becomes effectively of small size. With the inclusion or the reconstruction of the MHV, early graft function is satisfactory. The inclusion of the MHV or not in the donor's right lobectomy should be based on sound criteria to provide adequate functional liver mass for the recipient, while keeping the risk to the donor to the minimum [8].

De Villa et al. [9] described the Kaohsiung principle based on the donor-to-recipient body weight ratio, the volume of the donor's right lobe to the recipient's standard liver volume and the size of MHV tributaries from the anterior segment. Later, the Kyoto group, using the three-dimensional reconstructed images of the hepatic vascular anatomy, divided the right lobe graft morphologically into two types: one is a right hepatic vein dominant graft and the other is a MHV dominant graft [7, 10].

Regarding taking the right lobe graft with or without the MHV, we adopted a policy where we used to leave RHV within the donor guided by right lobe (mainly segments 5 and 8 have adequate venous drainage radiologically) and GRWR is 0.8 or more. If GRWR < 0.8 or MHV is the main drainage for the right lobe (provided that remaining liver volume is adequate for the donor, which is >35% of total liver volume) and there are no other donors available, we have to harvest the graft with MHV.

Our aim is to review the results of a policy for right lobe grafts without MHV and to analyze the methods of venous reconstruction related to outcome.

## 2. Patients and Methods

Over the period from January 2009 to January 2011, 40 patients underwent live donor liver transplant using a right lobe liver graft without the middle hepatic vein in the National Liver Institute, Menoufiya University, Egypt. This study has analyzed the results of these 40 cases.

Detailed data of all 40 donors and recipients were collected, tabulated, and analyzed with special concerns on clinical and demographic data of the donors including sex, age, body mass index (BMI) and relationship to recipient; history of medical diseases (especially hepatic diseases including Child-Pugh score and MELD score); examination: body build (weight and height) and abdominal examination for any scar of previous operation and any abnormalities by inspection and palpation.

The donor and recipient characteristics are given in Tables 1, 2. The selected donors were generally young (mean 24 years) with a low BMI (24.7). All donors were within the third degree relation of consanguinity as we generally do not accept unrelated donations for medicolegal issues, with sons presenting the highest percentage of 17 donors (42.5%) and cousins the lowest with 3 donors (7.5%).

All donors underwent liver biopsy. Four donors (10%) had steatosis and 2 donors (5%) had mild periportal fibrosis. These percentages are still within the accepted criteria for transplantation according to our policy and in most of the literature, besides these were the only available donors.

The recipients were aged from 24 to 60 years with a mean MELD of (16.3 + 4.4). Commonest etiology of the liver disease was HCV+/−HCC presenting up to (80%) Table 3. The graft-to-recipient weight ratios (GRWR) were above 0.8, ranged from a minimum of 0.84 to 1.9 (mean 1.09525 + 0.21).

Laboratory: complete blood picture, liver function tests, renal function tests, coagulation profile, blood sugar, electrolytes, HLA typing, crossmatching, tumor markers (AFP, CEA, CA19-9, and CA50), arterial blood gases, urine and stool analysis, and proteins S and C; serology: HBV DNA, antibody for HCV (PCR), HIV, CMV, EBV, varicella and rubella viruses, and liver biopsy.

Preoperative assessment of the donor anatomy: ultrasound and Doppler US: with special emphasis on liver parenchyma (steatosis and any lesion) and hepatic veins including distribution, number, caliber, and the presence of accessory veins. Triphasic computed tomography (CT): a serial coronal section view is especially useful to evaluate the hepatic veins variants and the volumetry of the graft and remnant liver.

## 3. CT Volumetry

A contrast material-enhanced CT examination of the abdomen was included in the evaluation and was required for the analysis of morphologic characteristics, the vascular status of the liver, and the evaluation of the hepatic parenchyma.

Magnetic resonance angiography and venography were done to evaluate the vascular variants of HA, HV, and PV.

### 3.1. MR Venography

**Figure 1:**
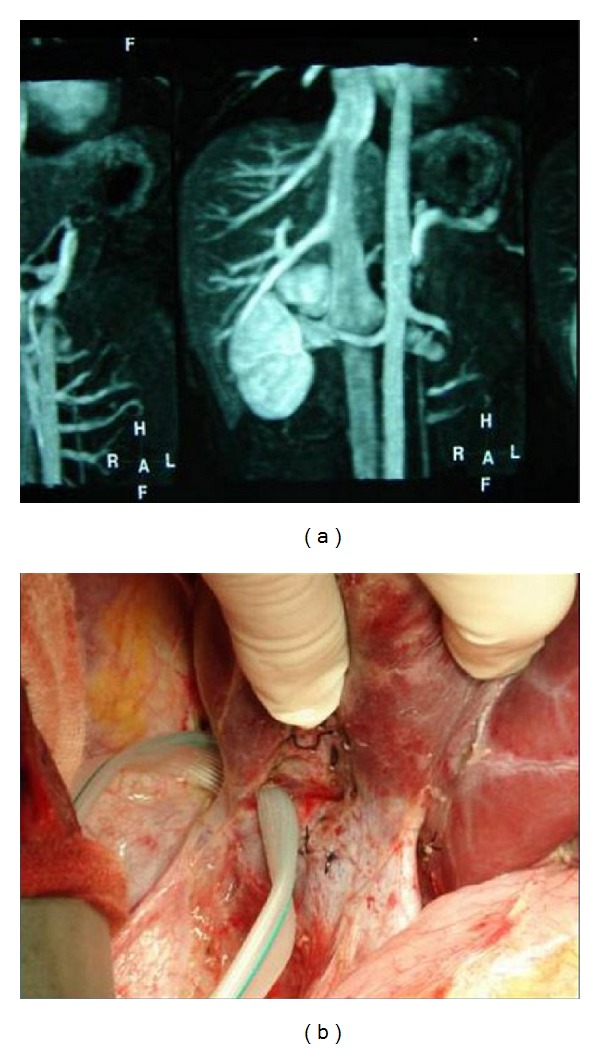
Big inferior right hepatic vein (IRHV) on MRV enography (a) and operative picture (b).

**Figure 2:**
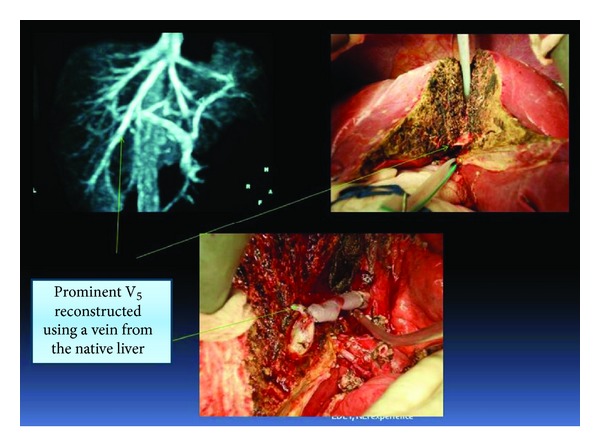
Big V5 vein in MRV: V5 in the graft during donor hepatotomy and after reconstruction in the recipient.

MRV was done using Philips Achieva 1.5 T machine. The following findings were recorded: (1) tributaries of the middle hepatic veins (MHV) including segments V and VIII veins; (2) the presence of accessory inferior right hepatic vein (IRHV) or superficial right hepatic vein (SRHV); (3) the variable entering patterns of the RHV, MHV, and IRHV into IVC, and (4) the diameter of the veins at their point of connection to the major veins. A comparison of the findings from the preoperative MR venography and the operative findings was made in an attempt to establish an accurate picture of the donor hepatic venous anatomy and plan the method of venoplasty, if at all required see Figures 1 and 2.

The intraoperative anatomical evaluation of biliary system is done by intraoperative cholangiography.

## 4. Surgical Procedures of LDLT

### 4.1. Donor Hepatectomy

LDLT was done through a bilateral subcostal incision with an upward midline extension. Intraoperative cholangiography via cystic duct was required to study the anatomy of the bile duct. Right hilar dissection was then performed to isolate the right hepatic artery, right portal vein, and right hepatic duct. Then the right lobe of the liver was rotated toward the left side for division of the ligaments on the right side of the liver and the minute hepatic venous branches. The liver was transected at a plane just to the right of the middle hepatic vein using an ultrasonic dissector. The transection plane was determined by intraoperative US and temporary occlusion of the right portal vein and right hepatic artery. When the transection approached the liver hilum, the right hepatic duct together with the surrounding Glission sheath was encircled. Then, the right hepatic duct was divided near the confluence of the hepatic ducts by scissors.

The transection was carried down to the junction of the right hepatic vein with the inferior vena cava. The right hepatic artery was then divided. The right hepatic vein was clamped at the junction with the inferior vena cava and divided. The stumps of the right portal vein and right hepatic vein were closed with continuous nonabsorbable sutures. The falciform ligament was sutured to the anterior abdominal wall. A drain was inserted into the right subphrenic cavity before wound closure.

### 4.2. Graft and Back Table

We perfused the grafts via the right PV with 2 liters of 4-degree histidine-tryptophan-ketoglutarate (HTK) solution which was also used to flush the biliary tract. The right hepatic vein orifice was inspected and all additional hepatic vein branches were identified and reconstructed, whenever indicated, using the criteria mentioned in the results section (Table 5). The size of all veins was measured using specific rulers. The grafts were weighed and graft to recipients weight ratio was calculated.

### 4.3. Operative Techniques in Recipients

The operative procedures were performed via chevron incision with upper midline extension.

When resecting recipients' liver, we attentively reserved posteriorhepatic inferior vena cava's (IVC) integrality, dissociated right hepatic vein cling to IVC, and reserved the orifices of right hepatic vein (RHV), along with the end axis enlarged IVC downward, making it suitable for donor's RHV and anastomosis. It was necessary to make ellipsed incision on suitable parts of IVC when the orifices of crassitude tributaries of right hepatic inferior vein or MHV were jointed with IVC by interpositioning the recipient portal vein, great saphenous vein, or cryopreserved cadaveric blood vessels. We adopted end-to-end anastomosis of grafts' right tributaries of PV to recipient's PV trunk, then opened blood flow in hepatic vein and PV, and ended nonhepatic phase period. Bypass was not used in all of our cases, as we only did partial IVC clamping. With loupe, we finished hepatic artery anastomosis and adopted end-to-end anastomosis of right hepatic duct to common hepatic duct, or Roux-en-Y choledochojejunostomy. If right hepatic duct had many tributaries and their caliber ≤2 mm, biliary tracts should be reconstructed under microscope. Splenectomy was performed at the same time if recipients suffered from splenomegaly and hypersplenism (blood platelet ≤ 30 × 10^9^/L). If PV pressure was >25 cm H_2_O, splenic artery ligation was performed for recipients in order to alleviate PV pressure.

Intraoperative Doppler US: a routine surveillance intraoperative US study entails grayscale assessment of the liver parenchyma and biliary tree and Doppler evaluation of the vasculature.

### 4.4. Postoperative Monitoring of Donor and Recipient

#### 4.4.1. Donor

Recovery: In ICU for two days. Medications: Receiving IV antibiotics, IV fluid for 3–5 days and then converting to oral feeding; pain killer and low molecular weight anticoagulant for 10 days s.c with daily followup by Doppler US, Lab (LFTs, RFTs, coagulation profile and CBS). Outcome: complications: acute rejection episodes, renal impairment, portal vein thrombosis, hepatic artery thrombosis, and biliary complications. Mortality: causes, rate, and analysis of survival. Discharge: 10–15 days after operation.

#### 4.4.2. Recipient

Recipient was in ICU for 1 week and then transferred for the transplant unit. Postoperative anticoagulant therapy: in most of the cases, heparin was used in more than 50 u/kg/day infusion. The dose was increased according to need to keep the INR between 2 and 3 for at least 10 days. Then the antiplatelets, persantin is given for one month. Postoperative Doppler US followup: Doppler US was used routinely for followup and was done twice for the first 10 days then once daily for detection of any early vascular and biliary problem and graft dysfunction.

We use broad spectrum antibiotics, mainly a combination of Meropenem and Metronidazole and then we do serial culture and sensitivity tests (from our infection control unit), then we might need to change regimen according to it. We also use Diclofenac (Fluconazole) as an antifungal prophylaxis for 7 days. In our antiviral regimen, we use Zovirax (Acyclovir) 200 mg tds starting from the 7th day postoperative till 6 months, and if there is CMV infection we use Ganciclovir (Cymevene vial) for 2 weeks. We used no prophylaxis for hepatitis. For immunotherapy, we use either Ciclosporin or Tacrolimus and steroids for 3 months. Regarding coagulopathy in recipients, especially in patients who had severe bleeding, we use blood, FFP, cryopreservation, and platelets transfusion and in some cases we use Factor 7 (Novo seven vial). We use prophylactic heparinization for 10 days, and then we shift for low molecular weight heparin (LMWH).

#### 4.4.3. Outcome

We compare both groups regarding laboratory findings (total bilirubin), overall hospital stay, three-month survival, and one-year survival. Long term outcome and survival analysis: calculation of outcome was done using Kaplan-Meier method.

#### 4.4.4. Statistical Analysis

Descriptive statistics were based on percentage for categorical data and on means, median, standard deviation, and range summarizing data distribution for continuous measures. Numerical data were presented as mean and standard deviation (SD). All data were analyzed using the SPSS package for windows. The following tests were used: Student's *t*-test, to test for significance when comparing the means of two sets of quantitative data, and the *P* (probability) value was considered to be of statistical significance if it was less than 0.05. Survival analysis was performed according to the Kaplan-Meier method from the date of surgery to that of death or event or to the most recent clinic visit.

## 5. Results

60% of grafts had a single right hepatic vein Table 4 and were directly anastomosed to the IVC. For all of our cases, we used to make longitudinal enlargement of the orifice of the right hepatic vein stump by the incising an anterior slit, down to its junction with the inferior vena cava (IVC) in order to guarantee wide and patent anastomosis.

**Figure 3:**
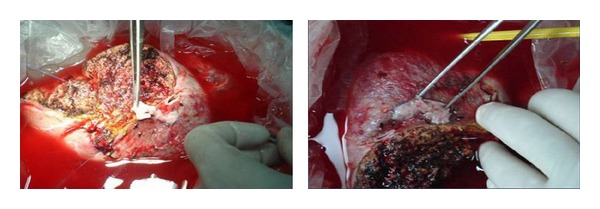
Multiple inferior hepatic veins (IHV) reconstructed into one opening using recipient PV graft.

**Figure 4:**
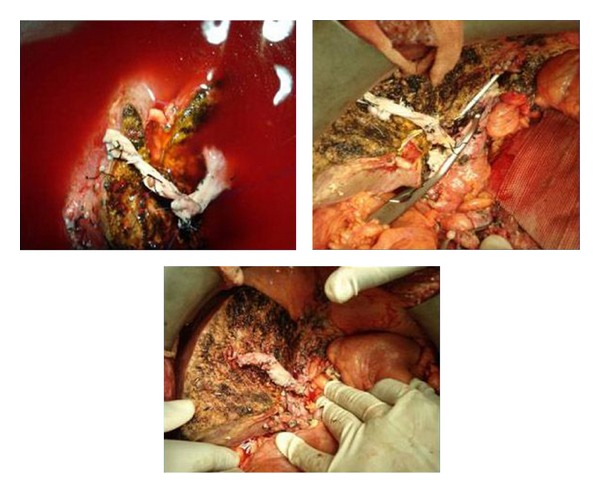
Multiple veins in graft: V5 reconstructed with PV graft with inferior right hepatic vein (IRHV) and RHV.

**Figure 5:**
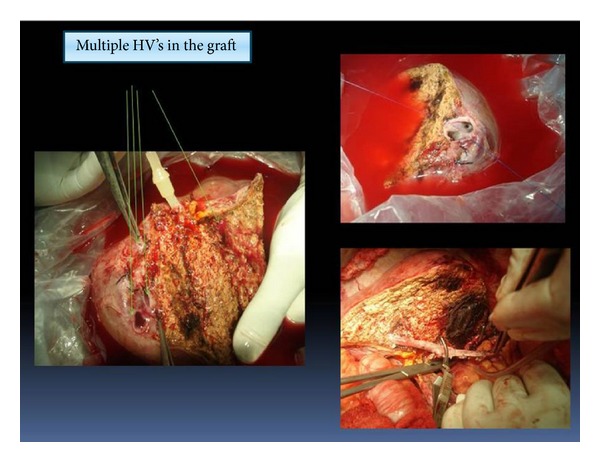
Multiple hepatic veins (HV) openings; V5, right hepatic vein (RHV), big posterior HV and inferior right hepatic vein (IRHV), and V5 reconstructed using PV graft to IVC.

At the time of transplant 16 of the 40 grafts (40%) were found to have more than 1 hepatic vein. The additional vessels and the methods of reconstruction are shown in Table 5. See Figures 3, 4, and 5.

Of the 16 patients with additional hepatic veins in the right lobe graft, 4 had interposition grafts and two venous patches to the anterior wall of RHV.

Special consideration had to be paid to accessory hepatic veins draining separately into the IVC, especially if the vein caliber is larger than 5 mm and there is a gap between the opening of this accessory vein and the main right hepatic vein. In that situation, it was necessary to make another IVC opening for extra separate anastomosis. That was the case in 14 cases out of the 16 cases with more than one single hepatic vein.

According to the source of the graft, there were 5 cases where we used portal vein of the recipient, and in the other one, we used recipient umbilical vein.

Two out of these six patients had acute rejection episodes in the early postoperative period; both of them had tolerated it and gradually improved with the proper postoperative care and adjusting the dose of immunotherapy.

The patency of the graft was followed up postoperatively with Doppler US for all of them, and they were all patent.

### 5.1. Postoperative Outcome

The cold ischemia time was significantly longer in those undergoing hepatic vein reconstruction Table 6 (mean = 68.75 (35–130) versus 51.25 (20–90)), *P* = 0.04688 as was the warm ischaemia time (mean 57.875 (30–80) versus 43.33 (25–75)), *P* = 0.00145. Finally, the HV anastomosis time in minutes had a mean = 34.6875 (15–65) for Group A and 17.70833 (15–30) for Group B with a *P* value of *P* = 0.0001.

The major complications in both groups Table 7 were mainly in the form of acute rejection episodes in 8 cases (20%); out of these eight patients, only one died early postoperative due to graft rejection, but the other seven patients tolerated it and gradually improved with the proper postoperative care and adjusting the dose of immunotherapy.

Renal impairment was 7 cases (17.5%); four out of them died early postoperative (almost all of them died within 2 months) due to the presence of other comorbidities in the form of HAT, biliary leak and sepsis, and heart failure, and one case with early graft dysfunction as well, while the other three passed it.

Portal vein thrombosis was in one case only (2.5%); portal vein thrombosis was diagnosed by color Doppler US; medical treatment in the form of increasing the dose of anticoagulants and changing from oral anticoagulant into injectable form was tried. Hepatic artery thrombosis was in 3 cases (7.5%); two of them had surgical reconstruction and did well and the third one died early postoperative as he had hepatic artery and portal vein thrombosis as well.

Biliary complications were in 16 cases (40%) with no significant difference between both groups; out of these sixteen cases, eight cases (20%) developed anastomotic leaks and the other eight cases developed biliary strictures. Out of these eight cases, six cases were managed conservatively, and two cases required surgical intervention.

### 5.2. Overall Morbidity and Survival (Table 8)

Again, when the comparison came to the outcomes in terms of laboratory findings (total Bilirubin on three-day levels and one-month levels), overall hospital stay, three-month survival, and one-year survival there were no significant differences between both groups, where the total bilirubin level after one month had a median of 0.8 +range (0.2–5.5) for Group A and a median of 1 + range (0.5–27) for Group B. The hospital stay in days had a median of 27 + range (13–51) for Group A and a median of 25 + range (5–84) for Group B.

The three-month survival was slightly better for Group A where it was 15 (93.75%) for Group A and 19 (79.16%) for Group B. The one-year survival as well was better for Group A with 14 patients out of 16 alive (87.5%) versus 17 patients out of 24 (70.83%) for Group B. The overall 1-year survival for our series was 31 cases out of 40 (77.5%).

## 6. Discussion 

In our study, all the forty grafts were right lobe grafts without the middle hepatic vein with exception only in two cases. The number of cases with more than one graft hepatic vein present intraoperative was sixteen cases (40%). Out of these sixteen cases, there were fourteen cases which actually required more than one hepatic vein anastomosis. All cases had only two vessel anastomoses (some of them after adjustment and refashioning of graft hepatic veins on the back table and this was to decrease warm ischemia time as much as possible).

Our results came in agreement with Marcos et al. 1999 who performed 25 right lobe living donor liver transplants without the MHV, with an excellent patient survival rate of 88% [11].

The Kyoto group, using the three-dimensional reconstructed images, divided the right lobe graft morphologically into two types: one is a right hepatic vein dominant graft in which the territory draining into the MHV is less than 40% of the right lobe graft, and the other is a MHV dominant graft [10]. Their indication for a right lobe graft with or without the MHV is based on dominancy of the hepatic vein, graft-to-recipient weight ratio, and remnant liver volume [7]. The group performed 217 right lobe LDLTs successfully according to this algorithm [12].

Right liver grafts with the MHV trunk (extended right lobe grafts) were first performed by the Hong Kong Group in 1996, as left lobe grafts from relatively small volunteer donors will not meet the metabolic demand of larger recipients [13]. Seven LDLTs, using this technique, were initially performed under high urgency situations. Although a high postoperative complication rate was reported (donors 29%, recipients 86%), the results are comparable to the best possible outcome in cadaveric transplantation for patients with similar status [14]. Meanwhile, another kind of right lobe liver grafts without the MHV (modified right lobe graft) emerged [11, 15] because the surgeons feared donor risk and important ethical issues. The extended right lobe grafts were too extensive as an operation for the donor [16, 17], and sufficient size of the remnant liver [18] as well as drainage of the segment 4 in the donor could not be guaranteed [5].

Some centers have introduced their experience in determining the extent of donor hepatectomy either with or without the MHV. de Villa et al. [9] described the Kaohsiung principle based on the donor-to-recipient body weight ratio, the volume of the donor's right lobe to the recipient's standard liver volume and the size of MHV tributaries from the anterior segment. This principle was applied in 25 living donor liver transplant operations and procured successful outcomes in both donors and recipients [12].

Adham et al. had 33 patients who received 34 cadaveric right split liver grafts. According to the type of recipient pairs (adult/adult or adult/child), the right liver graft was deprived of the MHV or not. They concluded that adult right SLT without the MHV is safe and associated with similar long-term results as compared with those of the right graft including the MHV, despite that early liver function recovered more slowly [19].

In our series, 40% of cases had atypical hepatic venous anatomy and showed more than one single right hepatic vein only within the right lobe graft. Fan Cheng et al.'s study showed that comparing MR venography with intraoperative surgical findings yielded clear visualization of the right, middle, and left hepatic veins in all cases, 100%. For those essential minor branches, which were equal to or larger than 5 mm in diameter, they obtained 88.2% accuracy [20]. The results of our study also illustrate that the MR venography has an accuracy of at least 87.5% of detecting accessory and minor hepatic veins and are in agreement with the literature supporting the use of MR venography for the definition of hepatic veins anatomy.

Since January 2006, Tashiro et al. have applied vascular closure staple technique successfully during liver transplantation in seven patients. They used this technique in reconstruction of the V8 and V5 tributaries in six and three patients, respectively, using the recipient's own middle hepatic vein. None of the patients experienced vascular complications and all had good venous flow postoperatively with excellent results [21].

To prevent RHV anastomotic stenosis, various methods of enlarging the RHV orifice have been introduced. Rather than constructing a standard end-to-end anastomosis between the orifices of the RHVs, Lee carried out simple enlargement of the orifice of the recipient vein by the creation of an anterior slit, down to its junction with the inferior vena cava (IVC), because the caliber of the recipient's RHV or caval orifice should be larger than the caliber of the liver graft's RHV for a wide and long-patent anastomosis [8]. That was the same strategy we adapted in our cases.

Marcos et al. introduced complete cavoplasty at the orifice of the RHV by creating an elliptical defect approximately 1.5 to 2.0 times the diameter of the donor RHV in the IVC [22].

Sugawara et al. introduced a new reconstruction method by which the anastomosis is lengthened by adding a venous patch. Long preservation of the recipient's RHV allowed the formation of a reservoir between the liver graft and the recipient's IVC. A transverse slit incision to the anterior wall of the RHV across the IVC orifice, and patch plasty with a U-shaped recipient portal vein, hepatic vein, or thick saphenous vein will enlarge the RHV orifice and allow reservoir formation [23]. We have also applied the same technique in two cases which had short right hepatic vein stump using a recipient portal vein.

The diamond shaped patch method carried out by the Tokyo group also allows for the widening of the RHV anastomotic orifice and for reservoir formation [23].

New strategies for HV reconstruction that would be tolerable to the compression of venous anastomotic sites by the regenerating liver graft were therefore suggested, one by the Tokyo group, using a cryopreserved large vein [24] and one by the Asan group, using an autogenous vein [25]. The Tokyo group introduced the double VC technique, using the cryopreserved VC to create a “common large opening” reconstruction when multiple major SHVs (caliber, ≥5 mm) were present. The Asan group has formulated a technique using the recipient's own autogenous vein instead of a cryopreserved VC. If the recipient portal vein showed no associated portal vein thrombosis and stenosis, the major SHVs were anastomosed to the interpositioned portal vein reservoir, which would prevent the compression occlusion of the anastomotic site by the enlarging liver graft [8].

Lee et al. [26] reported a good result of reconstruction of MHV tributaries of the anterior segment using the great saphenous vein. Cattral et al. [27] also described their successful use of the recipient's left portal vein as an interposition graft.

Regarding the type of the venous graft, we mainly used portal vein of the recipient in 5 cases, and in the other one, we used recipient's umbilical vein. Up to now, many types of vein grafts have been used for the reconstruction of the MHV, including the saphenous vein [6], umbilical vein, left portal vein, mainly from the recipient, and the inferior mesenteric vein and iliac vein, mainly from the donor [12]. Recently, some cryopreserved veins have been introduced for hepatic vein reconstruction [28]. This type of vein grafts might be the best way to keep outflow and make the reconstruction technically simple, but such vein grafts may have the problem of obstruction in the long-term observation period [29]. In WU Hong's study, they used the great saphenous vein as an interposition graft and formulated a strategy for reconstructing outflow in right liver grafts without the MHV [6].

Yu et al. (Hangzhou, China) in their institution, also mainly used the recipient's portal vein (main portal vein and its branch) as the interpositional MHV graft [12]. This kind of vein graft has several advantages over other vessels. Firstly, it is always available and easy to expose after the resection of the liver and eliminates the extensive dissection in the recipient or donor. Secondly, the suitable caliber, thick wall, and natural curvature of the portal vein can reduce the risk of thrombosis [27] after transplantation.

The European experience of adult LDLT summarized by Broelsch et al. [30] reported on 11 centers in 8 countries that performed 105 pediatric and 123 adult living donations, 111 of which were right lobe allografts. Recipient and allograft survival were 86% and 83%, respectively. Two large single center reports from France and Germany reported 1-year graft survival ranging from 75% to 85% [31, 32]. Marcos [33] reported that the survival rate of the first 20 recipients was 80% and improved to 95% in the next 20 recipients. We had very similar results with an overall 1-year survival for our series of 31 cases out of 40 (77.5%).

Bak et al. [34] of the University of Colorado reported 85% recipient survival in an initial series of 20 right lobe allografts. The primary causes of graft failure were primary nonfunction and vascular thrombosis. Their LDLT results in this series showed graft survival of 90.9%.

Adham et al. had 33 patients who received 34 cadaveric right split liver grafts. The first group (GI, *n* = 15) included grafts with only the right hepatic vein (RHV) outflow; the second (GII, *n* = 18) included grafts with both right and MHV outflows. The 2 groups were similar in patient demographics, initial liver disease, and donor characteristics. At one year, patient survival was 94% for both groups [19]. In our series, the one-year survival rates were better for Group A (reconstruction patients with more than one HV anastomosis, *n* = 16) 87.5%) with 87.5% versus 70.83% for Group B (patients with single HV anastomosis). All of the six patients with venous grafts are alive till now and doing well.

In Wu et al. series, recipients' survival rate was 89.1% (49/55) at median followup of 10 months (range, 1 to 26 months). Six patients (10.9%) died of small-for-size syndrome (1), renal failure (1), multiple organ failure (3) within 3 months after transplantation, and recurrent HCC (1) within 13 months after transplantation. The overall graft survival rate was 90.9% (50/55). Causes of graft failure were hepatic vein stricture (1), small-for-size syndrome (2), vascular thrombosis (1), and sepsis (1). One late death caused by tumor recurrence was not considered graft failure in this analysis [6]. In a study by Bin Liu's et al. consisting of 47 cases using right lobe graft without middle hepatic vein (MHV) and 3 cases using dual grafts (one case using two left lobe, 2 using one right lobe and one left lobe), among 50 adult recipients, 4 cases (8%) died postoperatively within 3 months. Their 1-year actual survival rate was 92% [6].

In summary, hepatic venous reconstruction in right lobe LDLT is technically challenging. A custom-made strategy in individuals may be necessary depending on whether significant MHV tributaries and major SHVs are present, although there is no consensus regarding the optional strategy for outflow reconstruction in LDLT without the use of the MHV. The most serious problem of adult LDLT without MHV is the obstruction of V5 or V8 outflow. The appropriate length of the reconstructed RHV is still controversial; a technique to secure an RHV anastomosis of adequate length and width may be a better option than a stretched, short anastomosis to prevent outflow obstruction. In our institute, we believe that adult LDLT is safely achieved with better outcome to both recipients and donors by harvesting the right lobe graft without MHV, provided that significant MHV tributaries (segments V and VIII more than 5 mm) are reconstructed, and any accessory considerable inferior right hepatic veins (IRHVs) or superficial RHVs are anastomosed.

## Figures and Tables

**Table 1:** 40 Donors data.

Donors data (40 donors)	
Age	Mean 24.55 + SD 5.35389
BMI	Mean 24.7275 + SD 3.73699
Sex	
Male	30 donors (75%)
Female	10 donors (25%)
Liver biopsy	
Normal	34 (85%)
Steatosis (maximum 10%)	4 (10%)
Very mild PPF	2 (5%)

**Table 2:** 40 Recipients data.

Recipients data (40 patients)	
Age	Mean 47.325 + SD 8.6362
Weight	Mean 78.2 + SD 12.20593
MELD score	Mean 16.3 + SD 4.40396
GRWR	Mean 1.09525 + SD 0.21211

**Table 3:** Indication for liver transplant.

Indication for liver transplant	Number (%)
HCV	19 (47.5%)
HCC	13 (32.5%)
Cryptogenic cirrhosis	5 (12.5%)
Alcoholic	1 (2.5%)
Budd chiari syndrome	1 (2.5%)
HBV	1 (2.5%)

**Table 4:** Results of preoperative MR venography and operative findings of venous anatomy.

No.	MRI venography	No. of HV in MRV	No. of graft HVs (intraoperative)	Use venous graft	Actual diameter of HV in mm (intraop.)	No. of HV anastomosis
*1 *	*RHV & V8 *	*2 *	*2 *	* Yes *	*22 & 9 *	*1 *
*2 *	*RHV & V5 *	*2 *	*2 *	*Yes *	*30 & 15 *	*2 *
*3 *	*RHV & IRHV *	*2 *	*2 *	*No *	*20 & 21 *	*2 *
**4**	**RHV**	**1**	**2**	**No**	**24 & 8**	**2**
**5**	**RHV & IRHV**	**2**	**3**	**Yes**	**29 & 16 & 11**	**2**
*6 *	*RHV & IRHV *	*2 *	*2 *	*No *	*33 & 18 *	*2 *
**7**	**RHV & V8 & IRHV**	**3**	**4**	**Yes**	**24 & 18 & 11 & 10**	**2**
*8 *	*RHV & V8 & V5 *	*3 *	*3 *	*No *	*31, 2 less than 5 mm *	*1 *
*9 *	*RHV & IRHV *	*2 *	*2 *	*No *	*26 & 13 *	*2 *
*10 *	*RHV & IRHV *	*2 *	*2 *	*No *	*26 & 13 *	*2 *
*11 *	*RHV & IRHV *	*2 *	*2 *	*No *	*36 & 15 *	*2 *
**12**	**RHV + MHV**	**2**	**3**	**Yes**	**23**—**20**—**14**	**2**
**13**	**RHV**	**1**	**2**	**No**	**28**—**14**	**2**
*14 *	*RHV & IRHV *	*2 *	*2 *	*No *	*35*—*18 *	*2 *
*15 *	*RHV & IRHV + MHV *	*3 *	*3 *	*Yes *	*20*—*27*—*21 *	*2 *
*16 *	*RHV & IRHV *	*2 *	*2 *	*No *	*32*—*15 *	*2 *
1	RHV	1	1	No	33	1
2	RHV	1	1	No	27	1
3	RHV	1	1	No	31	1
4	RHV	1	1	No	32	1
5	RHV	1	1	No	29	1
6	RHV	1	1	No	26	1
**7**	**RHV & V8**	**2**	**1**	**No**	**29**	**1**
**8**	**RHV & PRHV**	**2**	**1**	**No**	**33**	**1**
9	RHV	1	1	No	36	1
10	RHV	1	1	No	33	1
11	RHV	1	1	No	24	1
12	RHV	1	1	No	30	1
13	RHV	1	1	No	28	1
14	RHV	1	1	No	30	1
15	RHV	1	1	No	29	1
16	RHV	1	1	No	30	1
17	RHV	1	1	No	32	1
18	RHV	1	1	No	32	1
19	RHV	1	1	No	28	1
20	RHV	1	1	No	29	1
21	RHV	1	1	No	33	1
22	RHV	1	1	No	27	1
**23**	**RHV & V8**	**2**	**1**	**No**	**32**	**1**
24	RHV	1	1	No	33	1

*Highlighted (in bold) eight cases where the intra-operative findings were different from the pre-operative MRI venography recordings.

*Upper 16 italic cases: cases that had more than one graft hepatic vein intraoperatively.

**Table 5:** Hepatic venous variations in donor (actual intraoperative findings) and their reconstruction.

Hepatic venous variations in donor	Number of cases	Reconstruction	Method of reconstruction
Single IRHV	11	All	All IRHVs were anastomosed to IVC through an opening separate of that of RHV
2 IRHV	1	Yes	Interposition graft between 2 IRHVs into one opening into IVC
V5	1	Yes	Interposition graft between V5 and RHV into one opening into IVC
V8	1	Yes	Interposition graft between V8 and RHV into one opening into IVC
V5 + V8	1	Neither of them	—
2 IRHV + V8	1	Both	Interposition graft between 2 IRHVs into one opening into IVC, V8 to RHV

**Table 6:** Operative details.

Parameters	Group A (reconstruction patients with more than one HV anast.) (*n* = 16)	Group B (patients with single HV anast.) (*n* = 24)	*P* value
Cold ischemia time	Mean = 68.75 (35–130)	Mean = 51.25 (20–90)	0.04688
Warm ischemia time	Mean = 57.875 (30–80)	Mean = 43.33 (25–75)	0.00145
HV anastomosis time/min.	Mean = 34.6875 (15–65)	Mean = 17.70833 (15–30)	0.0001

The cold ischemia time was significantly longer in those undergoing hepatic vein reconstruction (mean = 68.75 (35–130) versus 51.25 (20–90)), *P* = 0.04688 as was the warm ischaemia time (mean 57.875 (30–80) versus 43.33 (25–75)), *P* = 0.00145. Finally, the HV anastomosis time in minutes had a mean = 34.6875 (15–65) for Group A and 17.70833 (15–30) for Group B with a *P* value of *P* = 0.0001.

**Table 7:** Major complications in both groups.

Complications	Group A (reconstruction patients with more than one HV anast.) (*n* = 16)	Group B (patients with single HV anast.) (*n* = 24)
Acute rejection episodes	4 (25%)	4 (16.6%)
Renal impairment	2 (12.5%)	5 (20.8%)
Portal vein thrombosis	1 (6.25%)	0 (0 %)
Hepatic artery thrombosis	1 (6.25%)	2 (8.3%)
Biliary complications	5 (31.25%)	11 (45.8%)

**Table 8:** Outcome in terms of laboratory findings (total bilirubin), overall hospital stay, three-month survival, and one-year survival.

Parameters	Group A (reconstruction patients with more than one HV anast.) (*n* = 16)	Group B (patients with single HV anast.) (*n* = 24)
Total bilirubin		
3-day level (mg/dL)	Median 3.25 + range (1.6–6)	Median 2.25 + range (0.3–10)
1-month level (mg/dL)	Median 0.8 + range (0.2–5.5)	Median 1 + range (0.5–27)
Hospital stay (days)	Median 27 + range (13–51)	Median 25 range (5–84)
Three-month survival	15 (93.75%)	19 (79.16%)
One-year survival	14 (87.5%)	17 (70.83%)
